# Sunlight driven NbSe_2_ photocatalyst for efficient degradation of Janus green and methylene blue dyes

**DOI:** 10.1038/s41598-025-05314-4

**Published:** 2025-11-04

**Authors:** Shivani R. Bharucha, Mehul S. Dave, Sunil H. Chaki, Tushar A. Limbani, Apurva C. Kadia

**Affiliations:** 1 N. V. Patel College of Pure and Applied Science, Charutar Vidya Mandal University, Gujarat, 388120 India; 2 P.G. Department of Physics, Sardar Patel University, Gujarat, 388120 India; 3 C.L. Patel Institute of Studies and Research in Renewable Energy, New Vallabh Vidyanagar, Charutar Vidya Mandal University, Gujarat, 388121 India

**Keywords:** NbSe_2_ semiconductor nanoparticles, Photocatalytic degradation, Janus green dye, Methylene blue dye, Environmental sciences, Materials science, Nanoscience and technology, Physics

## Abstract

With the world increasingly facing the challenge of dye pollution in water bodies, niobium diselenide (NbSe_2_) nanoparticles have emerged as promising photocatalysts for environmental remediation. In this study, NbSe_2_ nanoparticles were synthesized at room temperature, 70 °C, and 100 °C, and employed for the photodegradation of organic dyes Janus green (JG) and methylene blue (MB)—under natural sunlight. The synthesized nanoparticles were thoroughly characterized to confirm their phase composition, crystalline structure, morphology, and functional groups. Photocatalytic performance was evaluated through degradation efficiency and kinetic modeling using pseudo-first and pseudo-second-order reaction models. Results demonstrated that higher synthesis temperatures enhanced photocatalytic activity, with nanoparticles synthesized at 100 °C achieving > 99% degradation of JG and up to 98% for MB. The kinetic data fit best with the pseudo-first-order model, indicating a direct relationship between synthesis temperature and reaction rate constants. These findings underscore the pivotal role of synthesis temperature in tuning the photocatalytic properties of NbSe_2_ nanoparticles and affirm their potential in combating dye pollution through efficient solar-driven degradation processes.

## Introduction

In a world where rapid expansion of industrialization becomes the main contributor to water pollution, the issue is to provide and guarantee safe water for the entire environment^1,2^. A class of chemical molecules known as dyes is widely utilized in the culinary, printing, and textile industries^3^. About 1 to 20% of globally produced dyes are washed out during the dyeing process. They are extremely toxic and non-biodegradable and have a significant harmful impact on the environment^4–6^. The various organic dyes, which include methylene blue (MB), methyl orange, Janus green-B (JG), acridine orange (AO), and rhodamine-B (RB), are highly toxic and carcinogenic and are used for a variety of industrial and medical purposes, including treating methemoglobinemia and dying textiles^7,8^. MB and JG are extensively used in textile, pharmaceutical, and biological industries^9^. MB is commonly employed as a staining agent in microbiology and as a medication in various treatments^9^. On the other hand, JG is frequently used as a vital stain in histology and cytology. Despite their utility, both dyes contribute significantly to environmental pollution^8,10,11^. When discharged into water bodies, they can cause severe ecological harm, including toxicity to aquatic life, disruption of photosynthetic processes, and bioaccumulation in the food chain^10^.

Photocatalysis has developed as a potent method for environmental remediation, water purification, and sustainable energy generation. Photocatalysts in environmental remediation effectively decompose detrimental contaminants in air and water, transforming them into innocuous by-products like CO_2_ and H_2_O. Traditional treatment procedures such as adsorption and ozonation often prove inadequate in eradicating persistent organic pollutants owing to constraints in efficiency and selectivity^12^.

Advanced oxidation processes (AOPs) produce highly reactive hydroxyl radicals (·OH) on-site, providing an efficient method for the full mineralization of organic pollutants^12,13^. Semiconductor photocatalysis, a category of AOPs, has garnered significant interest owing to its environmental sustainability, economic efficiency, and reusability^14^. The benefits of semiconductor-based photocatalysts encompass: (1) minimal toxicity and cost-effectiveness; (2) adjustable electronic and optical characteristics by doping or nano-structuring; (3) enhancement of multi-electron transfer processes; and (4) consistent performance throughout several cycles^14–16^.

In recent decades, a variety of photocatalytic materials, including metal oxides, chalcogenides, sulphides, and non-metal doped nanostructures, have been investigated. NbSe_2_ is notable for its small bandgap, superior charge mobility, and chemical stability, enabling effective absorption of visible light and the formation of active species^17^. The environmental implications and potential toxicity of NbSe_2_ nanoparticles are important considerations for their application in photocatalysis. While NbSe_2_ is generally regarded as having lower toxicity compared to other semiconducting materials, such as titanium dioxide (TiO_2_) or zinc oxide (ZnO), it is still essential to evaluate its safety in environmental and biological contexts. The relatively low toxicity of NbSe_2_ makes it a promising candidate for photocatalytic applications, as it may pose fewer risks when released into the environment. However, the long-term effects of NbSe_2_ nanoparticles on ecosystems, including their potential for bioaccumulation and the generation of reactive oxygen species during photocatalytic processes, must still be considered.

Multiple techniques are available for the synthesis of NbSe_2_ nanostructures, such as physical vapour transport, chemical vapour deposition, and hydrothermal processes. Nonetheless, wet chemical synthesis offers a cost-effective and scalable option for photocatalytic applications. Factors including precursor content, pH, reaction temperature, and time significantly affects nanoparticles size, shape, and photocatalytic performance^18–20^.

A key innovative aspect of this study lies in demonstrating how increasing the synthesis temperature enhances the photocatalytic performance of NbSe_2_ nanoparticles under natural sunlight. As the synthesis temperature rises, notable improvements are observed in the structural, morphological, and optical properties of the material^21,22^, which contribute to more efficient degradation of JG and MB dyes. The NbSe_2_ photocatalyst synthesized at 100 °C exhibited the highest degradation efficiency, emphasizing the direct correlation between synthesis temperature and photocatalytic activity. This study provides valuable insights into optimizing NbSe_2_ for effective and sustainable dye removal in environmental remediation applications.

## Results and discussion

### ***Synthesized NbSe***_***2***_*** photocatalyst***

The synthesized NbSe_2_ photocatalyst nanoparticles exhibited distinct colour variation influenced by the synthesis temperature. The colour transits from brown at room temperature (RT) to light black and dark black as the synthesis temperature rises to 70 °C and 100 °C, respectively. This change in colour correlates with the structural and electronic modifications induced by the varying synthesis temperatures. At RT, the brown colour reflects a less dense arrangement of the NbSe_2_ and lower crystallinity, resulting in reduced light absorption. As the synthesis temperature increases, crystallinity improves and denser stacking enhances light absorption due to reduced bandgap and increased delocalization of charge carriers, imparting darker shades.

### Energy dispersive X-ray analysis

**Fig. 1:**
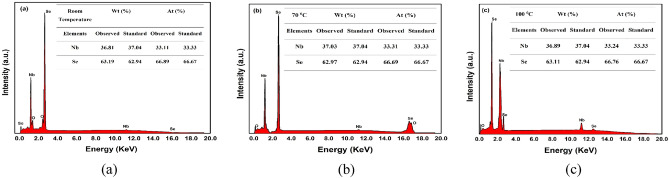
The elemental composition and observed atomic ratio of NbSe_2_ photocatalyst nanoparticles synthesized at (**a**) RT (**b**) 70 °C, and (**c**) 100 °C.

The compositional analysis of the synthesized NbSe_2_ photocatalyst nanoparticles was conducted using energy dispersive X-ray analysis (EDAX). The obtained stoichiometry confirms that pure NbSe_2_ was successfully synthesized, with no significant impurities detected. The EDAX peak profile and stoichiometric data, presented in Fig. 1, align with the expected elemental ratios for NbSe_2_, corroborating the purity and composition of the material. These findings are consistent with previously published results, further validating the reliability of the synthesis approach used^22,23^.

### X-ray diffraction profile

**Fig. 2:**
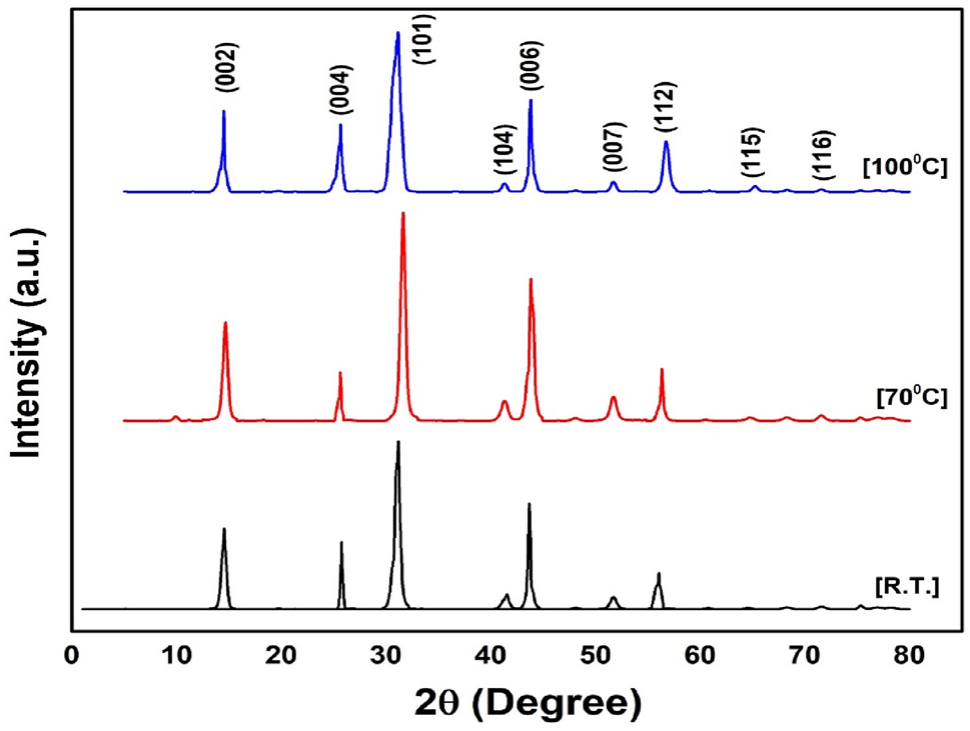
The XRD profile of NbSe_2_ photocatalyst nanoparticles synthesized at RT, 70 °C, and 100 °C.

The structural analysis of the synthesized NbSe_2_ photocatalyst nanoparticles was performed to confirm their crystallographic phase and purity. To confirm the phase and crystal structure of the synthesized NbSe_2_ nanoparticles, the resulting diffraction patterns were analyzed using Powder X software in reference to the standard JCPDS Card No. 018-0923, corresponding to the hexagonal phase of NbSe_2_. The results indicate that NbSe_2_ possesses the probable hexagonal crystal structure, characteristic of the 2H phase, with no detectable secondary phases or impurities. All detectable peaks, shown in Fig. 2, confirm the crystalline structure of NbSe_2_. The structural data aligns with standard crystallographic parameters *a* = *b* = 3.44 Å and *c* = 12.56 Å for NbSe_2_, confirming the successful synthesis of the pure phase material^23^.

### Scanning electron microscopy

**Fig. 3:**
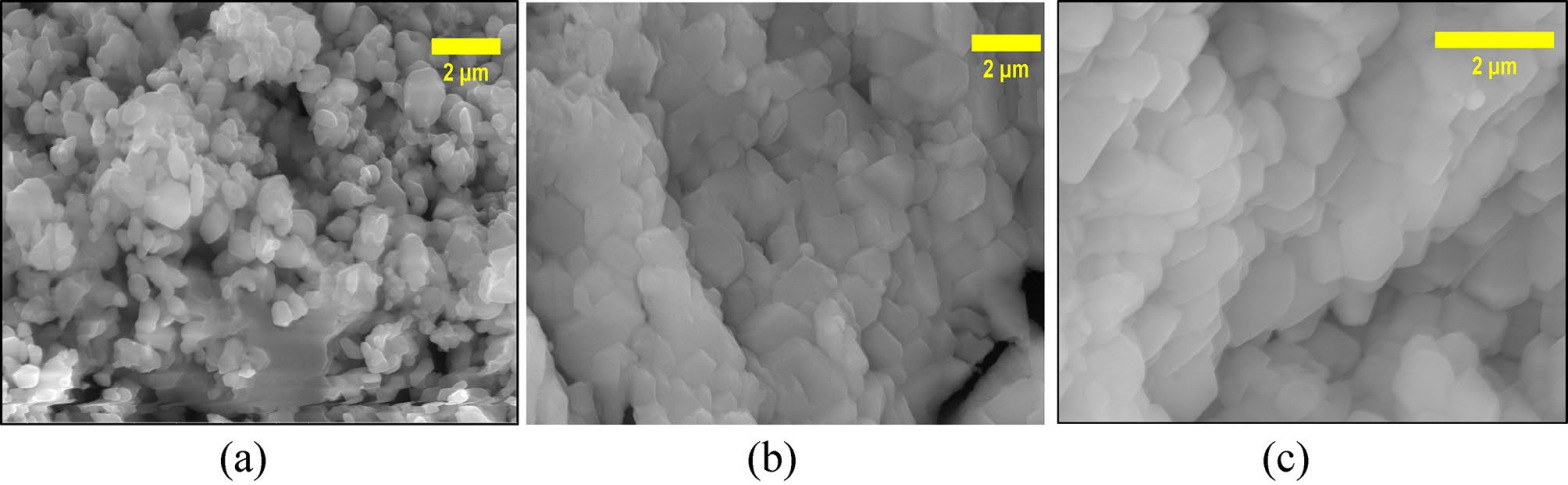
The surface morphology SEM images of NbSe_2_ photocatalyst nanoparticles synthesized at (**a**) RT, (**b**) 70 °C, and (**c**) 100 °C.

The SEM analysis reveals a clear correlation between the synthesis temperature and the morphology of the NbSe_2_ nanoparticles, demonstrating how temperature-driven morphological evolution influences their effectiveness in photocatalysis. At RT (Fig. 3a), significant agglomeration limits the active surface area and light absorption, reducing photocatalytic efficiency. At 70 °C (Fig. 3b), the reduced accumulation and more spherical morphology enhance light penetration and active site accessibility, leading to improved degradation performance. The 100 °C (Fig. 3c) synthesized nanoparticles has uniform size distribution, minimal agglomeration, and enhanced dispersion that maximizes the surface area and optimizes light absorption, resulting in superior photocatalytic activity. Thus, the temperature-dependent improvements in morphology directly contribute to the enhanced photocatalytic degradation efficiency of NbSe_2_ nanoparticles, with 100 °C yielding the most favourable structural properties.

### Transmission electron microscopy

**Fig. 4:**
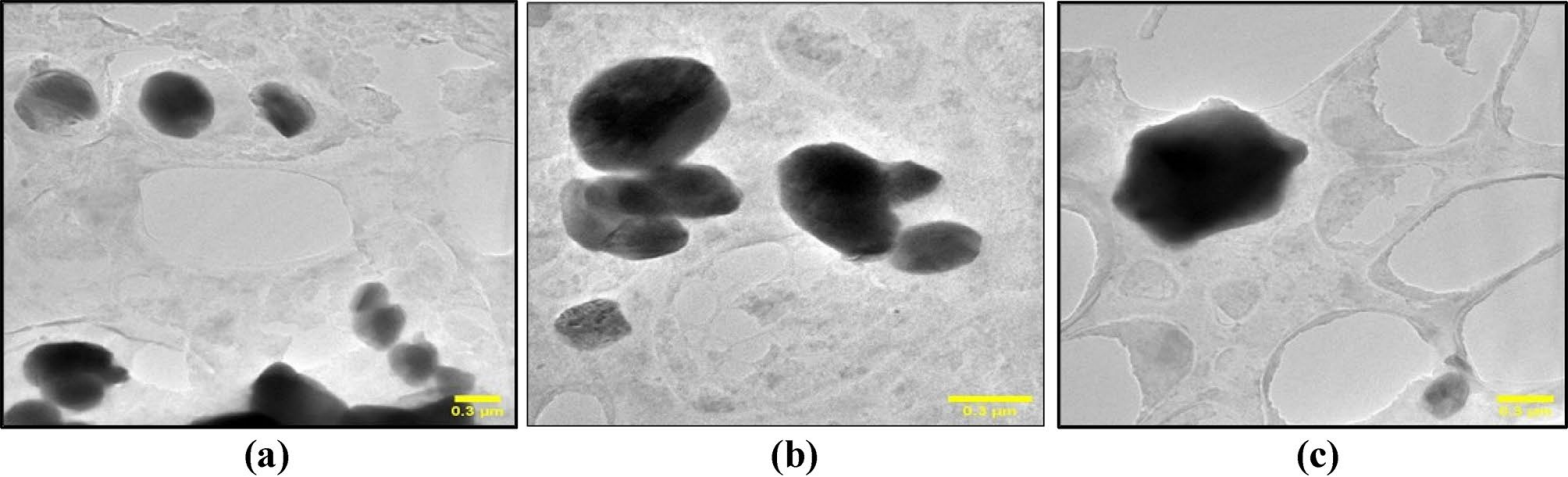
The surface topography TEM images of NbSe_2_ photocatalyst nanoparticles synthesized at (**a**) RT, (**b**) 70 °C, and (**c**) 100 °C.

TEM analyses the microstructure of NbSe_2_ nanoparticles synthesized via the sonochemical method at RT, 70 °C, and 100 °C, providing insights into how synthesis temperature affects their nanostructure topography. The morphology of NbSe_2_ nanoparticles synthesized at RT.

(Fig. 4a), 70 °C (Fig. 4b), and 100 °C (Fig. 4c), highlighting temperature dependent changes. At RT, the nanoparticles appear slightly agglomerated with hexagonal shapes, due to limited particles mobility and low energy, preventing proper dispersion. At 70 °C, the particles retain their hexagonal shape but show reduced agglomeration and improved dispersion, thanks to increased thermal energy allowing better separation. At 100 °C, the particles are well-dispersed with minimal agglomeration and the largest size, as the higher temperature promotes enhanced diffusion and growth while maintaining the hexagonal structure. The consistent maintenance of hexagonal morphology across all samples underscores the stability of the NbSe_2_ crystal structure under these synthesis conditions.

### Atomic force microscopy

**Fig. 5:**
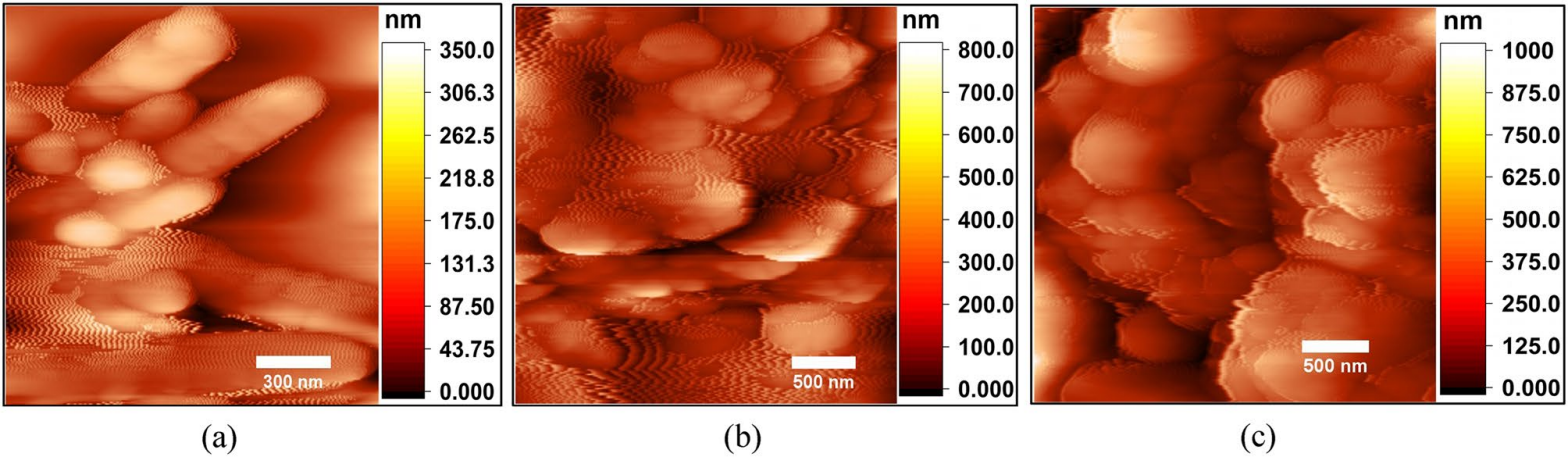
The surface topography AFM images of NbSe_2_ photocatalyst nanoparticles synthesized at (**a**) RT, (**b**) 70 °C, and (**c**) 100 °C.

The AFM analysis further confirms the influence of synthesis temperature on the morphological properties of NbSe_2_ nanoparticles, directly correlating with their photocatalytic performance. At RT (Fig. 5a), the rough surface and heterogeneous size distribution, coupled with significant agglomeration, limit the effective surface area and uniformity of the active sites, negatively impacting photocatalytic efficiency. The 70 °C (Fig. 5b) synthesized nanoparticles have smoother surface, improved size distribution, and reduced agglomeration, enhancing dispersion and accessibility of active sites for photocatalytic reactions. At 100 °C (Fig. 5c), the nanoparticles exhibit the smoothest surface and most uniform size distribution, minimizing agglomeration and maximizing the surface area for light absorption and pollutant interaction. These findings demonstrate that increasing synthesis temperature enhances the structural quality and dispersion of NbSe_2_ nanoparticles, significantly improving their photocatalytic degradation efficiency, with 100 °C providing the most optimized surface characteristics.

Table 1 summarizes key AFM parameters for NbSe_2_ photocatalyst, showing that average roughness (Ra) increases with temperature, from 23.34 nm at RT to 58.19 nm at 100 °C, indicating rougher surfaces due to enhanced grain growth. Skewness shifts from negative at RT (− 0.36) and 70 °C (− 0.52), reflecting surfaces with more valleys, to positive at 100 °C (1.02), indicating surfaces dominated by peaks. Kurtosis rises from 3.44 at RT to 12.66 at 100 °C, signifying increasingly sharp surface features.

**Table 1:** The AFM statistical parameter of NbSe_2_ photocatalyst nanoparticles synthesized at (a) RT, (b) 70 °C, and (c) 100 °C.

Sample	Average roughness (nm)	Skewness	Kurtosis
RT	23.34	− 0.36	3.44
70 °C	48.73	− 0.52	6.99
100 °C	58.19	1.02	12.66

### Fourier Transform Infrared Spectroscopy

**Fig. 6:**
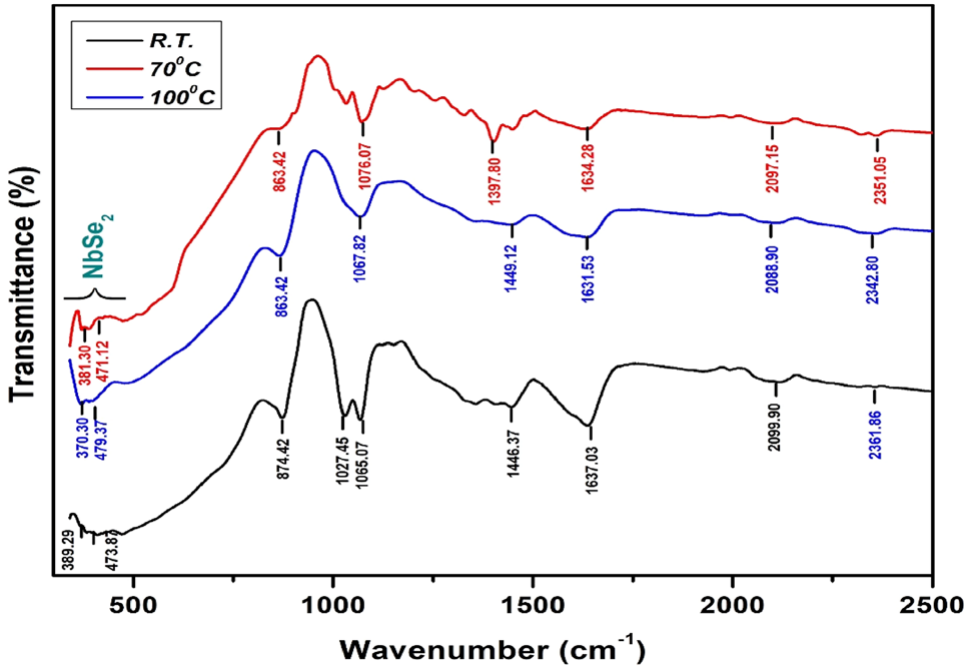
The FT-IR spectra of NbSe_2_ photocatalyst nanoparticles synthesized at RT, 70 °C, and 100 °C temperatures.

FT-IR analysis of NbSe_2_ photocatalyst nanoparticles synthesized at RT, 70 °C, and 100 °C reveals detailed insights into bonding environments, confirming the structural and electronic properties of the material (Fig. 6). The spectra highlight characteristic Nb–Se stretching and bending modes, with absorption bands around 500–600 cm^−1^ indicating strong Nb–Se bonding, which remains stable across synthesis temperatures. Bending vibrations in the 600–800 cm^−1^ range support a robust lattice structure essential for electronic stability. Peak broadening observed at 70 °C and 100 °C proposes increased disorder, likely due to phonon scattering or surface interactions, which may impact electronic transport by introducing localized states. In the higher range of 1000–1200 cm^−1^, bands linked to Se–Se or Se–Nb–Se configurations hint at complex bonding arrangements affecting charge transport. Temperature-related shifts in the Nb–Se stretching bands reflect thermal influences on bond environments, with changes in peak intensity correlating with particle growth and reduced agglomeration at elevated temperatures, affecting both the macroscopic and electronic properties of the nanoparticles.

### UV–Vis spectroscopy

**Fig. 7:**
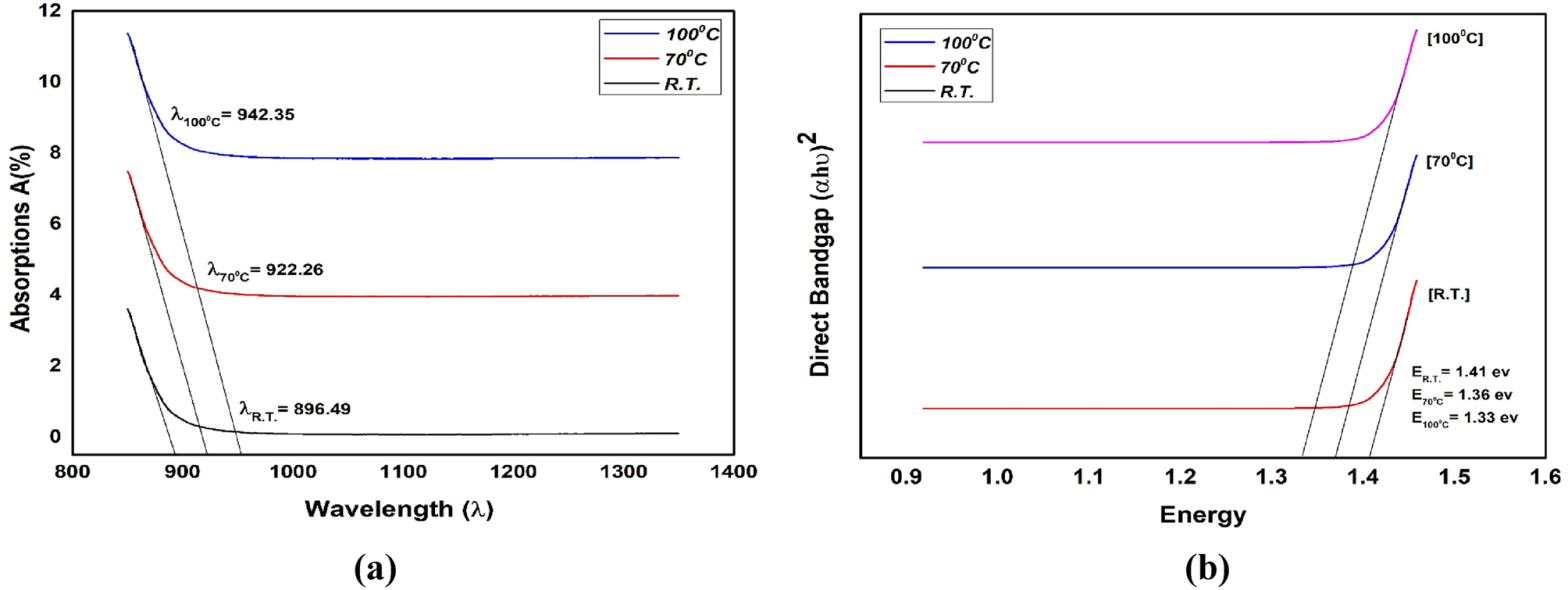
The absorption spectra and direct optical bandgap of NbSe_2_ photocatalyst nanoparticles synthesized at (**a**) RT, (**b**) 70 °C, and (**c**) 100 °C.

The optical bandgap analysis reveals a strong correlation between the bandgap energy of NbSe_2_ nanoparticles and their photocatalytic degradation performance. Figure 7 shows the absorption spectra and direct optical bandgap of NbSe_2_ photocatalyst nanoparticles synthesized at (a) RT, (b) 70 °C, and (c) 100 °C.

As the synthesis temperature increases, the redshift is observed in the absorption peaks, and the corresponding reduction in bandgap energy signifies a decrease in quantum confinement effects due to larger particle sizes. At RT, the smallest particle sizes and strongest quantum confinement yield a bandgap of 1.41 eV, which may limit photocatalytic activity by reducing the efficiency of light absorption in the visible range. At 70 °C, the bandgap decreases to 1.36 eV, improving light absorption and enhancing the generation of reactive species, thereby supporting better photocatalytic degradation. At 100 °C, the bandgap further reduces to 1.33 eV, allowing maximum utilization of the visible spectrum and increasing photocatalytic efficiency. This temperature-dependent decrease in bandgap energy facilitates broader light absorption, improving the photocatalytic performance of NbSe_2_ nanoparticles, with 100 °C producing the most optimal optical properties for degradation efficiency.

### Photocatalytic activity

The innovative use of NbSe_2_ nanoparticles as a photocatalyst was done with a study on the effect of photocatalysts’ synthesis temperature on degradation. However, its application as a photocatalyst for dye degradation has not been explored until now. This pioneering work investigates the potential of NbSe_2_ nanoparticles in degrading organic dyes under natural sunlight, offering a sustainable and efficient approach to environmental remediation. Additionally, the authors examined the effect of synthesis temperature on the photocatalytic performance of NbSe_2_ nanoparticles, providing insights into optimizing their preparation for enhanced dye degradation.

**Fig. 8:**
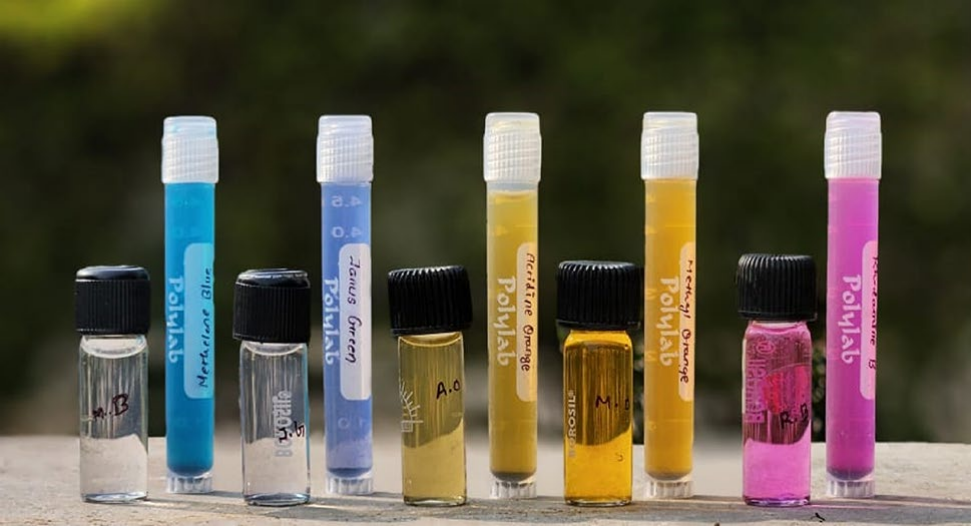
The experimental primary dye screening sample image of five different dyes using NbSe_2_ photocatalyst.

To assess the photocatalytic efficiency of NbSe_2_ nanoparticles, conducted a comprehensive screening of various dyes, including acridine orange (AO), rhodamine B (RB), methylene blue (MB), methyl orange (MO), and Janus green (JG). Figure 8 shows the experimental primary dye screening sample image of five different dyes using NbSe_2_ photocatalyst. These dyes were selected based on their widespread use in various industries and their known persistence in the environment. Among the tested dyes, MB and JG were chosen for further study due to their high degradation effect and significant environmental impact, and the challenges associated with their degradation^24–26^.

**Fig. 9:**
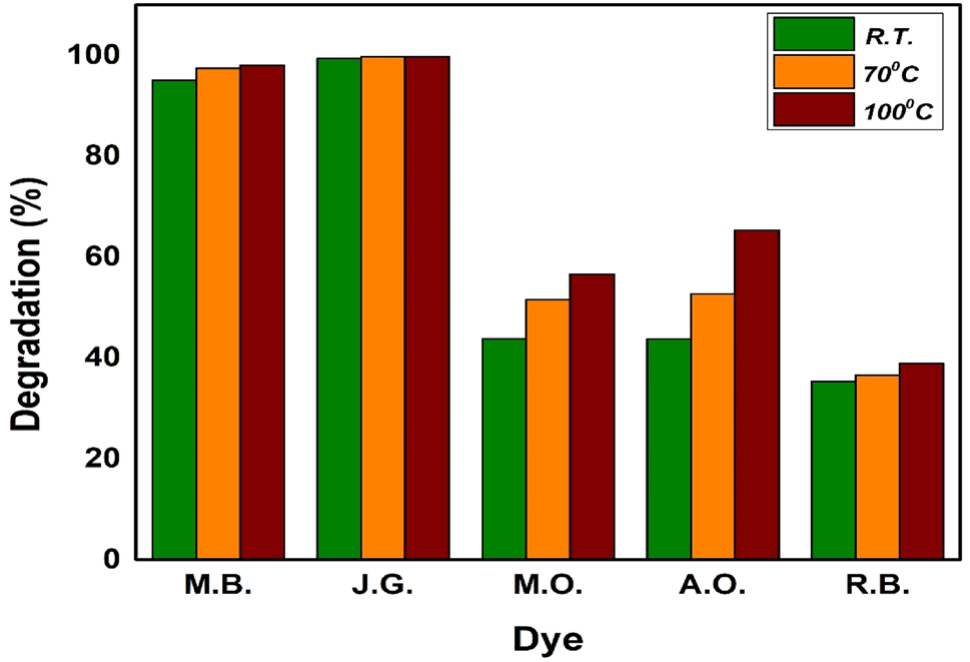
The dye screening of five different dyes employing NbSe_2_ photocatalyst nanoparticles for samples.

In Fig. 9, the degradation efficiency of different dyes using NbSe_2_ photocatalyst nanoparticles synthesized at RT, 70 °C, and 100 °C is illustrated in the bar graph. For MB, the degradation efficiencies for RT, 70 °C, and 100 °C are approximately 95%, 97%, and 98%, respectively, indicating a slight increase in efficiency with synthesis temperature of NbSe_2_ photocatalyst nanoparticles. JG shows a near-complete degradation for all three nanoparticles, with efficiencies consistently around 99%. MO degradation increases from about 60% with RT to around 80% with 100 °C synthesized nanoparticles. The AO also showed improvement; the degradation efficiency rose from approximately 55% for RT to 75% for 100 °C synthesized nanoparticles. For RB, the efficiencies are lower, ranging from about 40% for RT to 50% for 100 °C synthesized NbSe_2_ nanoparticles. These results demonstrate that the photocatalytic activity improves with successive NbSe_2_ photocatalyst nanoparticle samples (RT to 100 °C) across all dyes tested, with JG and MB showing the highest and most consistent degradation efficiency. Thus, authors further carried out an advanced intensive study on MB and JG dyes only.

### Proposed mechanism in degradation

**Fig. 10:**
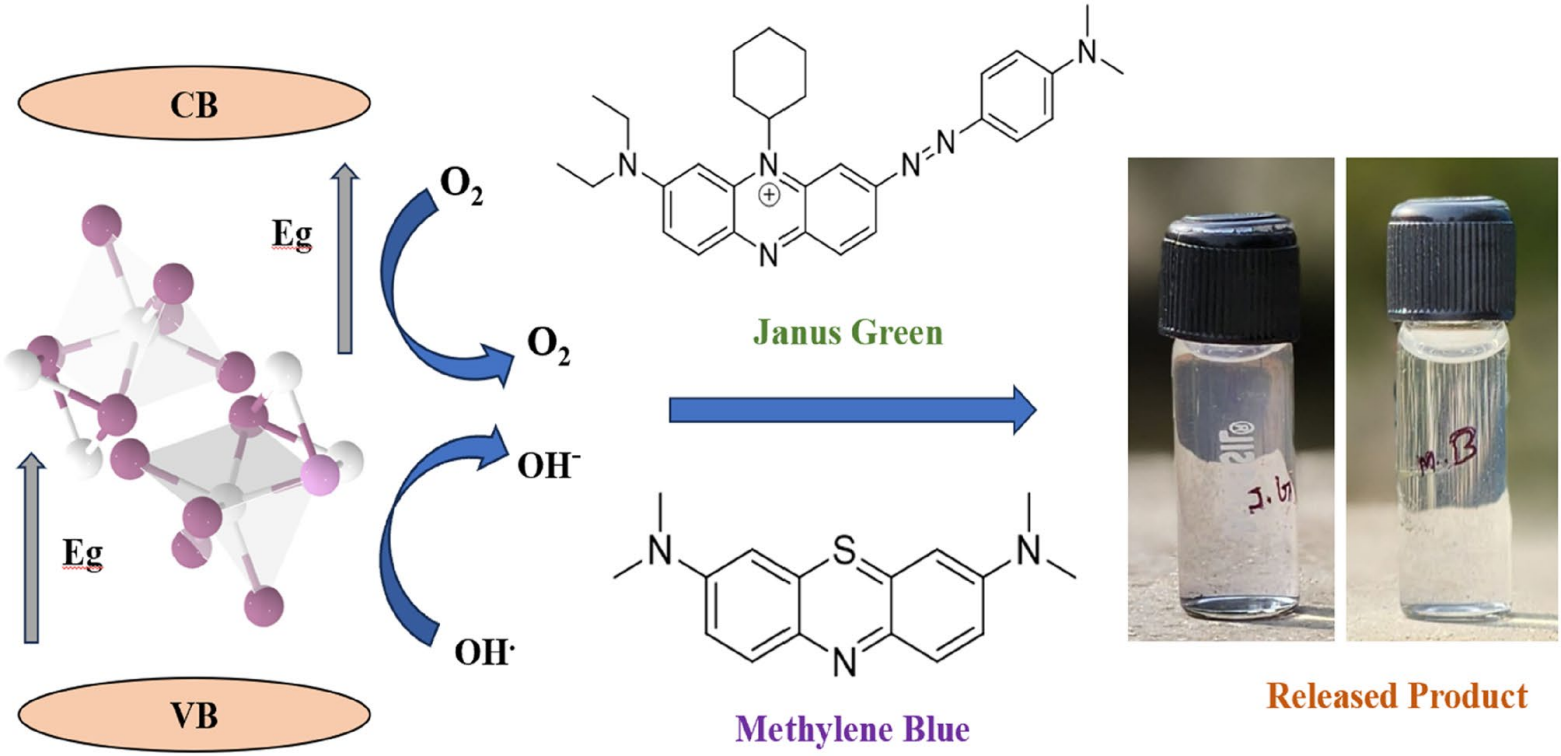
The proposed mechanism of JG and MB dyes degradation using NbSe_2_ photocatalyst of RT, 70 °C and 100 °C, respectively.

The proposed mechanism in schematic form for the photocatalytic degradation of JG and MB dyes using NbSe_2_ nanoparticles is shown in Fig. 10. The improved photocatalytic efficacy of NbSe_2_ nanoparticles is due to effective charge separation and transfer mechanisms during sunlight exposure. Upon light absorption, electrons in the valence band (VB) are elevated to the conduction band (CB), resulting in the formation of holes in the VB. The elevated conductivity and stratified architecture of NbSe_2_ promote swift electron movement, reducing charge recombination. Photogenerated electrons may engage in the reduction of oxygen to produce reactive oxygen species, including superoxide radicals (·O_2_ −), whereas holes facilitate the oxidation of water or hydroxide ions to provide hydroxyl radicals (·OH). These reactive species are pivotal in the breakdown of dyes. The combined impact of effective charge separation and reactive oxygen species production is essential for the reported photocatalytic activity.

The observed difference in degradation efficiency between JG and MB can be attributed to the distinct chemical properties and structures of the dyes. MB, being a cationic dye with a simpler structure, may interact more readily with the surface of NbSe_2_ nanoparticles, facilitating more efficient photocatalytic degradation. In contrast, JG, which has a more complex molecular structure, might exhibit weaker interactions with the photocatalyst, resulting in lower degradation efficiency. Furthermore, the electron transfer dynamics and reactive oxygen species generation during the photocatalytic process could vary for each dye, influencing their degradation rates. The size, charge, and molecular configuration of the dye molecules significantly impact their adsorption on the catalyst surface and subsequent degradation.

The synthesis temperature of NbSe_2_ nanoparticles also plays a critical role in their photocatalytic performance. Higher synthesis temperatures enhance the crystallinity, surface area, and defect states of NbSe_2_, leading to improved generation and transfer of reactive oxygen species, which boosts degradation efficiency. In contrast, lower synthesis temperatures result in reduced crystallinity and fewer active sites, diminishing the photocatalytic activity. This underscores the importance of optimizing the synthesis temperature to achieve maximum dye degradation efficiency.

### Factors affecting degradation

#### Effect of synthesis temperature

**Fig. 11:**
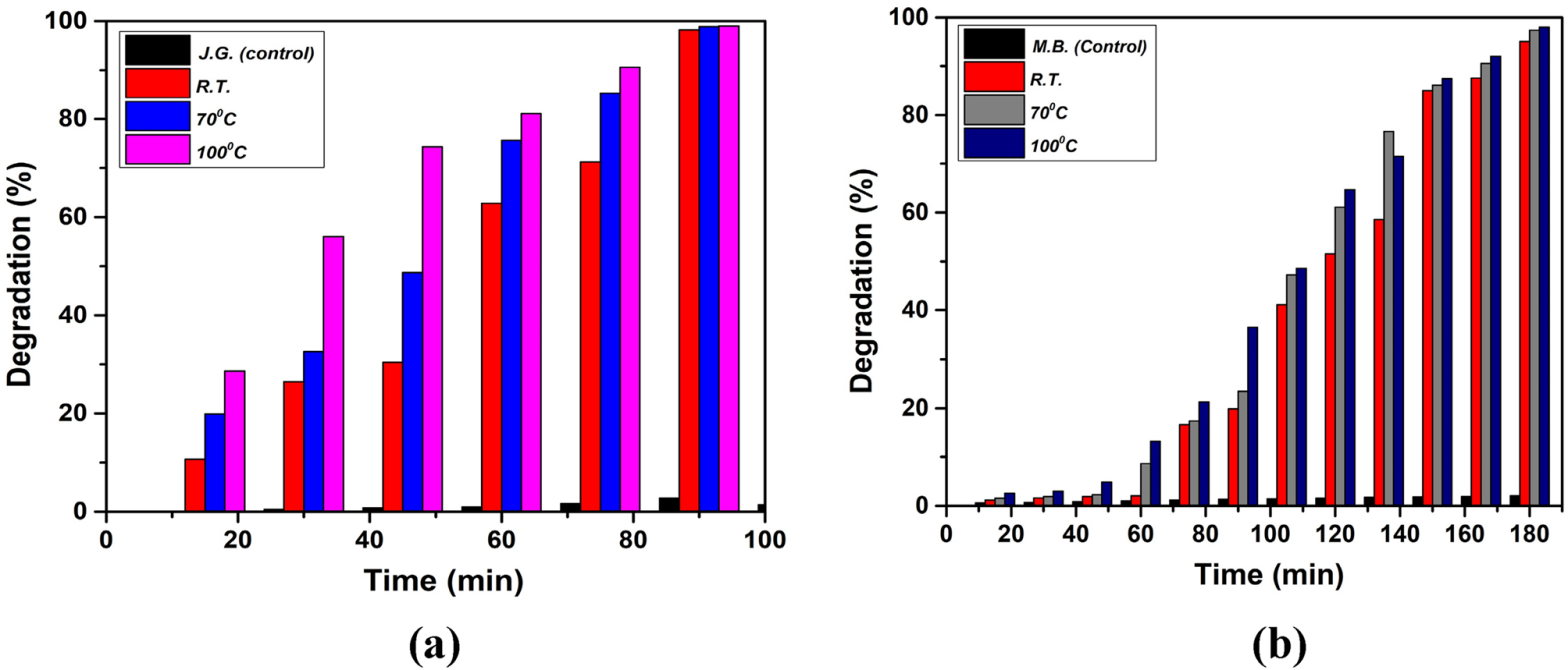
The dye screening of (**a**) JG and (**b**) MB dyes using NbSe_2_ photocatalyst nanoaprticles synthesized at RT, 70 °C, and 100 °C, respectively.

The photodegradation performance of NbSe_2_ photocatalyst nanoparticles synthesized at different temperatures (RT, 70 °C, 100 °C) was evaluated by monitoring the degradation of JG and MB under natural sunlight. Figure 11 presents the graphical data of JG and MB dye screening using NbSe_2_ photocatalyst nanoparticles synthesized at RT, 70 °C, and 100 °C. The degradation of JG was tracked over 100 min, revealing that the highest temperature of 100 °C synthesized photocatalyst nanoparticles achieves nearly 100% degradation within this timeframe, indicating superior photodegradation efficiency^27^. Nanocatalysts synthesized at a moderate temperature of 70 °C, followed closely, while synthesized at the lowest temperature of RT exhibited the least efficiency. This trend suggests that higher synthesis temperatures enhance the photocatalytic activity of NbSe_2_ nanoparticles, likely due to improved crystallinity and optimized electronic properties that facilitate more effective sunlight absorption and reactive oxygen species generation^28^.

Similarly, the degradation of MB was monitored for 180 min, with NbSe_2_ photocatalyst nanoparticles synthesized at 100 °C. It again demonstrated the highest photodegradation efficiency, achieving nearly 100% degradation within the timeframe. The photocatalyst nanoparticles synthesized at 70 °C showed high efficiency but required slightly more time to reach similar degradation levels, while photocatalyst nanoparticles synthesized at RT exhibited the lowest degradation rate. The consistent superior performance of 100 °C synthesized nanoparticles across both dyes underscores the importance of synthesis temperature in enhancing the photocatalytic properties of NbSe_2_ nanoparticles. The improved degradation rates for both JG and MB with increasing synthesis temperature suggest that optimal synthesis conditions are critical for maximizing the photocatalytic potential of NbSe_2_ nanoparticles^29^. The study demonstrates that the synthesis temperature of NbSe_2_ nanoparticles profoundly affects their photocatalytic performance in degrading organic dyes such as JG and MB. Higher synthesis temperatures result in nanoparticles with better crystallinity and enhanced photocatalytic properties, leading to more efficient degradation of these dyes under natural sunlight. Nanoparticles synthesized at highest temperature of 100 °C, consistently performed best, highlighting the importance of optimizing synthesis conditions to achieve superior environmental remediation outcomes.

The crystallite size of NbSe_2_ nanoparticles increases with synthesis temperature, as indicated by sharper and more intense diffraction peaks in the XRD analysis. Higher temperatures promote crystal growth, resulting in larger crystallites. This structural enhancement is correlated with improved photocatalytic degradation, as larger crystallites reduce electron–hole recombination, leading to more efficient charge separation and transfer. Therefore, the increase in crystallite size with synthesis temperature directly contributes to enhanced photocatalytic performance.

### Effect of dye concentration

**Fig. 12:**
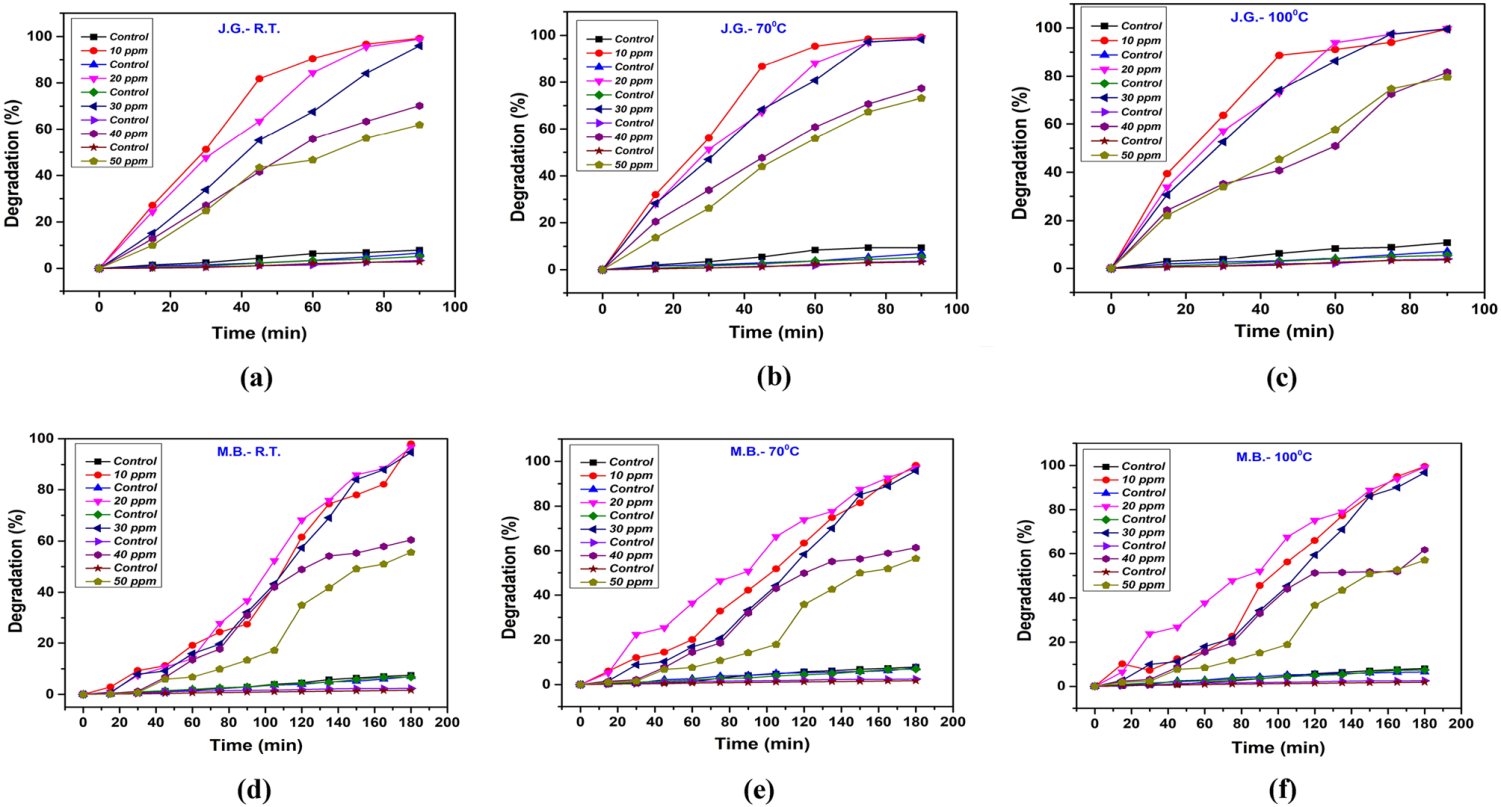
The photodegradation study with dye concentration of JG using NbSe_2_ photocatalyst of (**a**) RT, (**b**) 70 °C, and (**c**) 100 °C and of MB dye for (**d**) RT, (**e**) 70 °C, and (**f**) 100 °C.

The photodegradation efficiency of JG using NbSe_2_ photocatalysts synthesized at different temperatures was scrutinized to understand the impact of dye concentration on photocatalytic activity^30^. Figure 12a shows photocatalytic activity of the lowest temperature (RT) synthesized nanoparticles; it demonstrated high degradation efficiency at lower concentrations, achieving 99.16% degradation at 10 ppm within 100 min. However, as the dye concentration increased to 50 ppm, the degradation efficiency dropped to 61.88%. This indicates that RT photocatalyst can effectively degrade JG at lower concentrations, but its photocatalytic activity is hampered at higher concentrations due to limited reactive sites and suboptimal structural properties^31^. In contrast, 70 °C synthesized photocatalyst, Fig. 12b, show a slight performance improvement with a degradation efficiency of 99.21% at 10 ppm, and 73.16% at 50 ppm. The better crystallinity and improved light absorption of 70 °C contributed to its enhanced performance compared to RT, though it also experienced a decline in efficiency at higher concentrations^32^. Notably, Fig. 12c is of photocatalyst synthesized at 100 °C that exhibited the highest photodegradation efficiency across all dye concentrations. It achieved 99.51% degradation at 10 ppm and maintained a high efficiency of 79.52% at 50 ppm. The superior performance of 100 °C synthesized photocatalyst can be attributed to its optimal crystallinity and electronic properties, which enhances sunlight absorption and facilitates the efficient generation of reactive oxygen species^33^.

This sustained high photocatalytic activity even at higher dye concentrations underscores the importance of optimizing synthesis conditions. The comparative analysis reveals that the synthesis temperature of NbSe_2_ nanoparticles significantly affects their photocatalytic efficiency. The sample of 100 °C consistently outperformed RT and 70 °C, demonstrating that higher synthesis temperatures lead to nanoparticles with superior photocatalytic properties. Thus, the study emphasizes the critical role of synthesis temperature in tailoring the structural and electronic properties of NbSe_2_ nanoparticles to achieve effective photocatalytic performance for environmental remediation applications.

The photodegradation efficiency of MB using NbSe_2_ photocatalysts synthesized at different temperatures reveals crucial insights into how synthesis conditions impact photocatalytic performance^29^. Photocatalyst synthesized at lowest temperature of RT, Fig. 12d, demonstrate a high degradation efficiency of 97.92% at a lower dye concentration of 10 ppm within 120 min. However, this efficiency significantly decreased to 55.56% at a higher concentration of 50 ppm. This indicates that RT, while effective at lower concentrations, suffers from reduced photocatalytic activity at higher concentrations due to limited reactive sites and suboptimal structural properties^34^. In Fig. 12e, photocatalyst synthesized at moderate temperature of 70 °C showed an improved performance. At 10 ppm, it achieved a degradation efficiency of 98.13%, near similar to RT photocatalyst, but it maintained a higher efficiency of 56.40% at 50 ppm. This improvement can be attributed to better crystallinity and more effective light absorption in 70 °C, allowing it to handle higher concentrations of MB more efficiently, though still experiencing a decline at elevated concentrations^35^. Figure 12f is of sample synthesized at 100 °C, it exhibited the best performance across all dye concentrations. At 10 ppm, it achieved a degradation efficiency of 99.58%; even at 50 ppm, it maintained a high efficiency of 57%. The superior performance of 100 °C synthesized photocatalyst is due to its optimal crystallinity and electronic properties, which enhance sunlight absorption and facilitate the efficient generation of reactive oxygen species, sustaining high photocatalytic activity even at higher dye concentrations^18^.

The comparative analysis shows that higher synthesis temperatures result in NbSe_2_ nanoparticles with superior photocatalytic properties, as evidenced by the consistently better performance of 100 °C synthesized nanoparticles compared to RT and 70 °C. While RT and 70 °C exhibited decreasing degradation efficiencies at higher dye concentrations, 100 °C maintained robust performance, underscoring the importance of optimizing synthesis conditions to enhance the photocatalytic potential of NbSe_2_ nanoparticles. These findings highlight the critical role of synthesis temperature in optimizing the structural and electronic properties of NbSe_2_ nanoparticles, making them highly effective for environmental remediation applications, particularly in degrading organic pollutants like MB across various concentrations.

### ***Effect of NbSe***_***2***_*** catalyst dosage***

**Fig. 13:**
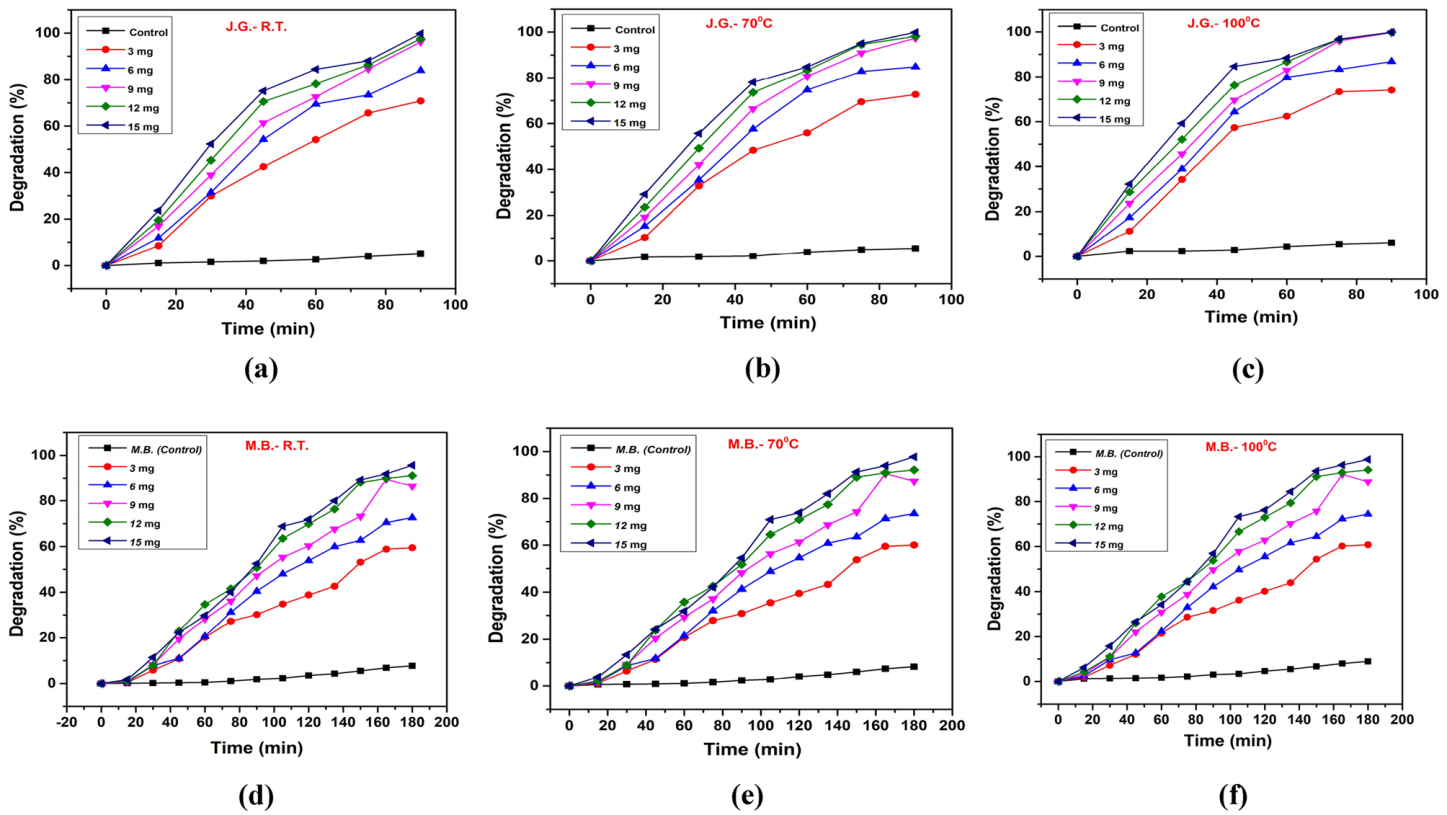
The effect of NbSe_2_ photocatalyst nanoparticles dosage on JG dye for photocatalyst nanoparticles synthesized at (**a**) RT, (**b**) 70 °C, and (**c**) 100 °C and for MB dye for photocatalyst nanoparticles synthesized at (**d**) RT, (**e**) 70 °C, and (**f**) 100 °C.

The Fig. 13 illustrates the degradation efficiency of NbSe_2_ photocatalysts nanoparticles synthesized at RT, 70 °C, and 100 °C for the degradation of JG and MB dyes under varying catalyst dosages. The different dosages taken for study were 3 mg, 6 mg, 9 mg, 12 mg, and 15 mg. The effect of catalyst dosages (3 mg, 6 mg, 9 mg, 12 mg, and 15 mg) on the photodegradation of JG dyes was investigated, showing significant differences in degradation efficiency. As the catalyst dosage increased, the degradation of JG improved notably.

For RT, Fig. 13a, the degradation efficiency increased with increasing catalyst dosage, reaching a maximum of 89% degradation at 15 mg dosage after 90 min. Lower dosages (3 mg and 6 mg) exhibited lower efficiencies of 42% and 59%, respectively. For 70 °C synthesized photocatalyst, Fig. 13b, the degradation efficiency improved significantly. The highest dosage (15 mg) achieved 98% degradation, whereas lower dosages (3 mg and 6 mg) showed moderate degradation of 51% and 68%, respectively, in the same duration. The 100 °C synthesized photocatalyst, Fig. 13c, the photocatalytic performance was slightly enhanced compared to 70 °C. The 15 mg dosage achieved 99% degradation within 90 min, while lower dosages followed a similar trend, with 3 mg showing 55% degradation.

These results indicate that higher catalyst dosages provide more active sites for the degradation process, enhancing the breakdown of JG molecules^36^. The plots shows marginal improvement between the highest dosages, it suggests a possible saturation point where additional catalyst does not significantly enhance degradation efficiency^37^. This study demonstrates that optimizing catalyst dosage is crucial for maximizing the photodegradation of dyes using NbSe_2_ photocatalysts nanoparticles.

The influence of varying catalyst dosages (3 mg, 6 mg, 9 mg, 12 mg, and 15 mg) on the photodegradation of MB dye was assessed, revealing substantial enhancements in degradation efficiency with increased catalyst amounts. In case of RT synthesized photocatalyst nanoparticles, Fig. 13d, the degradation efficiency for MB dye was slower and required a longer duration of 180 min to reach optimal levels. The maximum efficiency of 87% was achieved at a 15 mg dosage, while lower dosages showed 40% (3 mg) and 57% (6 mg) degradation. The 70 °C synthesized photocatalyst nanoparticles, Fig. 13e, showed significant increase with 15 mg achieving 96% degradation in 180 min. Lower dosages (3 mg and 6 mg) resulted in 48% and 65% degradation, respectively. The 100 °C synthesized photocatalyst nanoparticles, Fig. 13f, reached its degradation efficiency peak, as 15 mg dosage achieved a maximum of 98% degradation, while the 3 mg dosage exhibited 50% degradation over 180 min. The data indicate that higher catalyst dosages provide more active sites for the photodegradation process, facilitating the breakdown of MB molecules more effectively. As observed in the plots, the marginal gains between the highest dosages suggest a point of diminishing returns, where additional catalyst does not proportionally enhance degradation^38^. This study underscores the importance of optimizing catalyst dosage to achieve maximum photodegradation efficiency of MB dye using NbSe_2_ photocatalysts nanoparticles.

The results demonstrate that both synthesis temperature and catalyst dosage play critical roles in enhancing the photocatalytic degradation of dyes. Increasing the synthesis temperature improves the crystallinity, dispersion, and active surface area of the NbSe_2_ photocatalysts nanoparticles, resulting in higher degradation efficiencies. Additionally, higher catalyst dosages provide more active sites for the photocatalytic reaction, further boosting dye degradation. The nanoparticles samples synthesized at 100 °C exhibited superior degradation performance for both dyes, with JG dye showing nearly complete degradation (99%) at 15 mg dosage within 90 min, while MB dye required 180 min to achieve similar degradation (98%).

### Kinetic absorptions

**Fig. 14:**
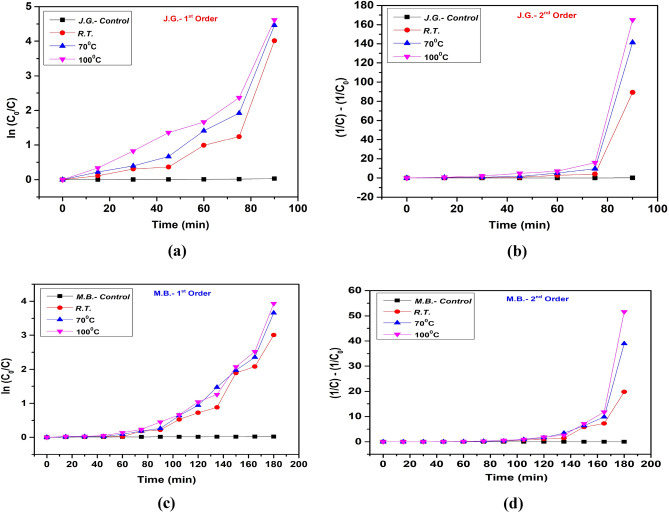
The (**a**) 1st order and (**b**) 2nd order kinetics of JG dyes and (**c**) 1st order and (**d**) 2nd order kinetics of MB dyes using NbSe_2_ photocatalyst for samples of RT, 70 °C, and 100 °C.

The Fig. 14 depicts the kinetics of a reaction or process at different temperatures, analyzed through first and second-order models. Each set of graphs can be discussed in terms of the specific behaviours and trends observed.

The First-order graph, Fig. 14a, for JG displays the natural logarithm of the concentration ratio *ln*(C/C_0_) over time for a reaction controlled by first-order kinetics at different temperatures and a control. The control line shows minimal change, suggesting negligible reaction progress in its absence. For the reaction conditions: RT gradually increases, indicating a slow reaction rate^39^. While at 70 °C, the rate rises noticeably compared to RT, indicating enhanced reactant activity^19^. The 100 °C condition exhibits the steepest slope, signifying the fastest reaction rate among the tested synthesis temperatures.

The second-order graph, Fig. 14b, of JG illustrates the reciprocal of the concentration difference over time, suitable for reactions following second-order kinetics. The trend shows initially the control remains flat, showing almost no reaction progress. The reaction for RT synthesized samples progresses, albeit slowly. As the samples synthesis temperature increases to 70 °C and 100 °C the rate of reaction increases dramatically particularly towards the later time points, as shown by the sharp upward trajectory of the graph lines. This sharp increase particularly for samples synthesized at higher temperatures suggests that as the reactants decrease, the effect of the concentration on the reaction rate becomes more pronounced, in line with the characteristics of second-order kinetics where the rate is proportional to the square of the reactant concentration^40^.

Figure 14c shows the first-order graphs for MB degradations. The *ln*(C/C_o_) again measures the natural logarithm of the concentration ratio over time. In MB also initially the control line shows a steady negligible increase. For RT synthesized samples the reaction progresses steadily, suggesting a moderate reaction rate^41^. The increase of sample synthesis temperature to 70 °C and to 100 °C significantly accelerates the reaction rate, evidenced by steeper slopes^42^. These trends affirm that synthesis temperature is a critical factor in enhancing the reaction rate, likely by providing the necessary energy to surpass the activation energy threshold more efficiently^43^.

While in the second-order kinetics, Fig. 14d, the control shows minimal change, similar to the first set. The reaction for RT synthesized nanoparticles shows a slow but steady increase in rate. The plots for 70 °C and 100 °C temperature synthesized nanoparticles show a significant increase after the 140-min mark, with samples synthesized at 100 °C showing a particularly steep rise, suggesting a very fast reaction rate at higher concentrations and temperatures. This indicates that for MB, the reaction rate is highly sensitive to both temperature and reactant concentration, which is typical for second-order reactions where both factors play a significant role^25^.

Across both sets of reactions for JG and MB, the data consistently demonstrate that increased reaction time dramatically enhance the reaction rates, aligning with theoretical expectations for chemical kinetics. The control experiments serve as important baselines to highlight the impact of synthesis temperature and time of reaction. The results validate the kinetic models chosen for describing the reaction under these specific conditions, providing a robust framework for predicting and controlling the behaviour of such reactions in practical applications.

The kinetic adsorption of the photocatalytic degradation rate constant of different samples was evaluated using the pseudo-first and second-order models. The percentage of photo-degradation and the rate constants for each sample are provided in Table 2.

**Table 2:** The first and second-order kinetic rate constants with correlation of photocatalytic degradation by NbSe_2_ photocatalyst nanoparticles.

Sample	% of photo-degradation	Rate constant			
		K_1_	R_1_^2^	K_2_	R_2_^2^
JG-Control	5.921	2.855 × 10^−4^	0.864	4.756 × 10^−4^	0.861
JG-RT	99.342	0.035	0.614	0.661	0.298
JG-70 °C	99.671	0.042	0.738	1.062	0.318
JG-100 °C	99.752	0.044	0.838	1.263	0.345
MB-Control	2.059	1.023 × 10^−4^	0.954	1.066 × 10^−4^	0.955
MB-RT	95.056	0.014	0.749	0.067	0.451
MB-70 °C	97.425	0.017	0.772	0.118	0.361
MB-100 °C	98.043	0.018	0.762	0.149	0.325

The control samples, Control-JG and Control-MB, exhibited very low photo-degradation efficiencies of 5.921% and 2.059%, respectively, indicating minimal degradation of the target compounds without the photocatalyst. In contrast, the photocatalysis of JG (JG-RT, JG-70 °C, JG-100 °C) demonstrated remarkably high photo-degradation efficiencies, all above 99%, showcasing the photocatalyst’s effectiveness. Similarly, the MB (MB-RT, MB-70 °C, MB-100 °C) also showed high photo-degradation efficiencies, ranging from 95.06 to 98.04%, indicating significant but slightly lower photocatalytic activity compared to the JG samples.

Whereas, the first-order rate constant (K_1_) values for the JG and MB are significantly higher than those of the control samples, confirming enhanced degradation kinetics in the presence of the photocatalyst. Among the JG, the rate constant increases from JG-RT (0.035 min^−1^) to JG-100 °C (0.044 min^−1^), indicating an improvement in photocatalytic efficiency, with JG-100 °C showing the highest R_1_^2^ value of 0.838. The MB samples follow a similar trend, with increasing K_1_ values from MB-RT (0.014 min^−1^) to MB-100 °C (0.018 min^−1^) and moderate R_1_^2^ values, with MB-70 °C having the highest R_1_^2^ value of 0.772. The second-order rate constant (K_2_) values for JG samples are significantly higher than those for the MB samples, indicating different kinetic behaviours for the two sets of samples^44^. However, the R_2_^2^ values are relatively lower for the second-order kinetic model compared to the first-order model, suggesting that the first-order kinetic model provides a better fit for the degradation data.

### Compared with other photocatalysts

Compared to conventional photocatalysts such as TiO_2_, ZnO, and CdS, the NbSe_2_-based photocatalyst demonstrated competitive and, in some cases, superior photocatalytic activity under identical experimental conditions as mentioned in Table 3. While TiO_2_ and ZnO typically require UV light activation due to their wide band gaps, NbSe_2_ exhibits visible-light-driven photocatalytic behaviour owing to its narrow band gap and metallic conductivity, which enhances charge carrier mobility and reduces recombination rates. The proposed study demonstrated that NbSe_2_ nanoparticles synthesized at optimal temperatures degraded MB and JG more quickly and efficiently than many traditional photocatalysts. The degradation efficiencies reached up to 98.04% within 120 min and 99.75% within 100 min. These results highlight NbSe_2_ as a promising next-generation photocatalyst under visible light.

**Table 3:** Comparison of the present work with other traditional photocatalysts.

Photocatalyst	Target dye	Light source	Degradation efficiency (%)	Time (min)	Remarks	References
TiO_2_ (P25)	MB	UV light (365 nm)	~ 98	180	Requires UV, not effective under visible light	^45^
ZnO	MB	UV light	~ 94	150	Efficient under UV, less stable in water	^46^
rGO-Ag	JG	UV light	~ 95		Require UV and photocatalyst synthesis using waste dry cell battery rod and *Corchorus olitorius* extract as GO precursor and reducing agent	^47^
Sr-TiO_2_	JG	Visible light	~ 92	120	Efficient under UV light with Sr-doped	^48^
NbSe_2_ (this work)	MB	Natural Sunlight	~ 98.04	180	High activity under visible light	
NbSe_2_ (this work)	JG	Natural Sunlight	~ 99.75	100	Good degradation efficiency, less studied dye	

## Materials and methods

### Reagents and materials

All the chemicals used for the synthesis of NbSe_2_ photocatalyst were of analytical grade and used without any filtration. Niobium pentachloride dihydrate (NbCl_5_·2H_2_O) [minimum assay 99%, Alfa Assar, United States], hydrochloric acid (HCl) [minimum assay 35%, HiMedia Laboratories Pvt. Ltd., Mumbai, India], tri-ethanol amine (TEA) [minimum assay 98%, Sisco Research Laboratories (SRL) Pvt. Ltd., India], sodium selenite (Na_2_SeO_3_) [minimum assay 98.5%, HiMedia Laboratories Pvt. Ltd., Mumbai, India] and hydrazine hydrate (N_2_H_4_) [Sigma Aldrich Pvt. Ltd., India] were used.

### ***Synthesis of NbSe***_***2***_*** photocatalyst nanoparticles influenced by synthesis temperature***

In this study, the synthesis of NbSe_2_ photocatalyst nanoparticles was carried out with notable modifications compared to previous work^49^. These modifications encompassed changes in the selection of precursor chemicals, adjustments in the synthesis temperature, and the adoption of an alternate synthesis method. The rationale behind these modifications was to tailor the material properties and optimize the photocatalytic activity for our specific research objectives. First, a 1 M solution of niobium ions source is prepared by dissolving 1.35 g NbCl_5_·2H_2_O in 5 mL 50% dilute HCl by continuous magnetic stirring. After 20 min of continuous stirring, 2.79 mL of 3.75 M of TEA is added to the solution. The TEA works as a complex agent. The solution turns transparent from a milky white colour. To the above solution, 13 ml of already prepared 2 M Na_2_SeO_3_ is added. The 2 M Na_2_SeO_3_ is prepared by dissolving 3.45 g of Na_2_SeO_3_ and 3 mL of hydrazine hydrate in 10 mL de-ionized water. Here, the 2 M Na_2_SeO_3_ solution acted as a source of selenium ions. The hydrazine hydrate helped to separate the Se ions from the solution. The final solution is made 50 mL by adding double distilled water under continuous stirring. The stirring is continued for 2 h, during this, the solution turns into a dark orange colour and then turns out to be black colour. The ultimate solution is kept isolated under sonication to allow the nanoparticles to set into the bottom of the beaker. By following the same process, the NbSe_2_ photocatalyst nanoparticles were synthesized at three different temperatures, RT, 70 °C, and 100 °C respectively. The black colour precipitate settled at the bottom of the beaker was filtered using Whatman filter paper (Grade 5) and given several washes of double-distilled water to obtain the NbSe_2_ nanoparticles. After multiple washings, all the samples were dried at 100 °C temperature for 4 h, and finally, the black-coloured NbSe_2_ nanoparticles were obtained.

### Characterization

All the sonochemically synthesized NbSe_2_ photocatalyst nanoparticles, prepared at varying synthesis temperatures, were comprehensively characterized to understand their structural, compositional, morphological, optical, and chemical properties. The elemental composition and surface morphology were investigated using energy-dispersive X-ray spectroscopy (EDAX) attached to a high-resolution field emission scanning electron microscope (FE-SEM, Nova Nano SEM-450, FEI Ltd.), operated at SICART, Vallabh Vidyanagar. EDAX confirmed the elemental presence of niobium and selenium, while FE-SEM provided insights into the particles’ morphology and dispersion quality. Surface topography and nanoscale roughness were further examined through atomic force microscopy (AFM) at CSMCRI, Bhavnagar. The optical characteristics were analyzed using a UV–Vis-NIR spectrophotometer (Lambda 19, Perkin Elmer), which enabled the determination of absorption profiles and estimation of optical band gaps. Chemical bonding and functional groups were identified through Fourier Transform Infrared (FT-IR) spectroscopy in the range of 300 to 4000 cm⁻^1^, revealing information on surface chemistry and potential interactions relevant to photocatalytic activity.

The crystalline structure and phase purity of the synthesized NbSe_2_ nanoparticles were determined through X-ray diffraction (XRD) using a Bruker D8 Advance diffractometer with CuKα radiation (λ = 1.5432 Å). The obtained diffraction patterns were analyzed using PowderX software and compared with standard reference data from the JCPDS database. The diffraction peaks matched well with the JCPDS card no. 018-0923, confirming the formation of the hexagonal (2H) phase of NbSe_2_.

The transmission electron microscopy (TEM) analysis of the nanoparticles was carried out employing TEM. Surface topography and nanoscale roughness were further examined through atomic force microscopy (AFM) at CSMCRI, Bhavnagar. Chemical bonding and functional groups were identified through Fourier Transform Infrared (FT-IR) spectroscopy in the range of 300 to 4000 cm⁻^1^, revealing information on surface chemistry and potential interactions relevant to photocatalytic activity. The optical characteristics were analyzed using a UV–Vis-NIR spectrophotometer (Lambda 19, Perkin Elmer), which enabled the determination of absorption profiles and estimation of optical band gaps.

### Photocatalytic degradation experiments

Natural sunlight was used as the irradiation source for the photocatalytic experiments. To ensure consistent light exposure, the solar intensity was measured using a pyranometer, and all experiments were conducted within an irradiation range of 900 to 1000 W/m^2^. The tests were performed under clear sky conditions during similar daytime hours. Ambient temperatures during the experiments ranged from 37 to 40 °C. These measures were taken to minimize variability and ensure reliable assessment of photocatalytic performance.

Stock solutions of JG and MB were prepared by dissolving the respective dyes in distilled water. The initial experiments utilized a dye concentration of 30 ppm and a catalyst dosage of 15 mg. For further analysis, dye solutions of five different concentrations (10 ppm, 20 ppm, 30 ppm, 40 ppm, and 50 ppm) were prepared. The experiments were conducted with varying catalyst dosages of 3 mg, 6 mg, 9 mg, 12 mg, and 15 mg.

### Experimental setup

A series of 100 mL dye solutions at the desired concentrations was prepared. NbSe_2_ nanoparticles (RT, 70 °C, and 100 °C) were added to each dye solution at the specified catalyst dosages.

### Photodegradation process

The reaction mixtures were exposed to natural sunlight for a predetermined period (90 min for JG and 180 min for MB). At regular intervals of 15 min, aliquots of the reaction mixtures were withdrawn for analysis.

### Reusability of the experiment

The reusability of NbSe_2_ nanoparticles was assessed by several photocatalytic degradation cycles. The findings demonstrated steady performance with no substantial decline in efficiency, demonstrating robust stability and longevity. These results validate the capability of NbSe_2_ as a reusable and dependable photocatalyst for sustainable applications.

### Analytical measurements

The concentration of the dye in the supernatant was determined using UV–Vis spectrophotometry by measuring the absorbance at specific wavelengths corresponding to the maximum absorption of JG and MB. The degradation efficiency (%) was calculated using the initial and final absorbance value by the following Eq. (1);

1 $${\text{Degradation}}\;\left( \% \right) = \frac{{A_{0} - A_{t} }}{{A_{0} }} \times 100$$

where A_0_ presents the initial absorbance and A_t_ is the absorption at time t. The pseudo-first and second-order kinetic adsorption (R^2^) and rate constant K (min^−1^) were determined by using the following Eqs. (2) and (3), respectively.

2 $$\ln \left( {\frac{{C_{t} }}{{C_{0} }}} \right) = - kt$$

3 $$\frac{1}{{C_{t} }} = \frac{1}{{C_{0} }} + k_{2} t$$

where C_0_ and C_t_ are concentrations at the beginning and at a certain time, t is the irradiation time.

## Conclusion

The NbSe_2_ nanoparticles synthesized at RT, 70 °C, and 100 °C exhibited significant photocatalytic activity in degrading JG and MB dyes under natural sunlight, with synthesis temperature crucial in determining photocatalytic efficiency. The 100 °C NbSe_2_ sample achieved the highest degradation rates, with 99.2% for JG and 97.6% for MB within 100 and 180 min, respectively. The kinetic analysis confirmed that the photocatalytic degradation of both dyes followed pseudo-first-order kinetics, with the highest rate constant observed in the case of the 100 °C sample (0.0452 min^−1^ for JG and 0.0318 min^−1^ for MB). Characterization through XRD, SEM, TEM, AFM, and UV–VIS Spectroscopy demonstrated a stable crystalline structure and confirmed the decreasing bandgap that contributed to enhanced photocatalytic activity. The study conclusively shows that synthesis temperature not only influences the structural and morphological properties of NbSe_2_ but also its functional capability in environmental remediation. This work proposes that NbSe_2_ synthesized at optimized conditions, particularly at 100 °C, can be a highly efficient photocatalyst for degrading dye pollutants, offering a sustainable solution for wastewater treatment applications.

However, the mechanism of photocatalytic degradation, though initially explored using trapping experiments, remains partially understood due to the qualitative nature of the tests. A more in-depth investigation, such as electron spin resonance spectroscopy or advanced scavenger studies, is required to comprehensively elucidate the role of reactive species. Additionally, this study did not investigate the degradation by-products or perform toxicity assessment factors which are critical for real-world environmental applications. These important aspects will be addressed in future studies to strengthen the practical relevance of the proposed photocatalytic system.

## Data Availability

All data generated or analyzed during this study are included in this published article.
